# Vesiculation from *Pseudomonas aeruginosa* under SOS

**DOI:** 10.1100/2012/402919

**Published:** 2012-02-14

**Authors:** Reshma Maredia, Navya Devineni, Peter Lentz, Shatha F. Dallo, JiehJuen Yu, Neal Guentzel, James Chambers, Bernard Arulanandam, William E. Haskins, Tao Weitao

**Affiliations:** ^1^Department of Biology, The University of Texas at San Antonio, One UTSA Circle, San Antonio, TX 78249, USA; ^2^Pediatric Biochemistry Laboratory, The University of Texas at San Antonio, San Antonio, TX 78249, USA; ^3^Department of Biology, The University of Texas at San Antonio, San Antonio, TX 78249, USA; ^4^Department of Chemistry, The University of Texas at San Antonio, San Antonio, TX 78249, USA; ^5^RCMI Proteomics, The University of Texas at San Antonio, San Antonio, TX 78249, USA; ^6^Protein Biomarkers Cores, The University of Texas at San Antonio, San Antonio, TX 78249, USA; ^7^Center for Research and Training in the Sciences, The University of Texas at San Antonio, San Antonio, TX 78249, USA; ^8^Division of Hematology and Medical Oncology, Department of Medicine, Cancer Therapy and Research Center, University of Texas Health Science Center at San Antonio, San Antonio, TX 78229, USA

## Abstract

Bacterial infections can be aggravated by antibiotic treatment that induces SOS response and vesiculation. This leads to a hypothesis concerning association of SOS with vesiculation. To test it, we conducted multiple analyses of outer membrane vesicles (OMVs) produced from the *Pseudomonas aeruginosa* wild type in which SOS is induced by ciprofloxacin and from the LexA noncleavable (*lexAN*) strain in which SOS is repressed. The levels of OMV proteins, lipids, and cytotoxicity increased for both the treated strains, demonstrating vesiculation stimulation by the antibiotic treatment. However, the further increase was suppressed in the *lexAN* strains, suggesting the SOS involvement. Obviously, the stimulated vesiculation is attributed by both SOS-related and unrelated factors. OMV subproteomic analysis was performed to examine these factors, which reflected the OMV-mediated cytotoxicity and the physiology of the vesiculating cells under treatment and SOS. Thus, SOS plays a role in the vesiculation stimulation that contributes to cytotoxicity.

## 1. Introduction

A Gram-negative bacterium, *Pseudomonas aeruginosa *has emerged as a prevalent nosocomial pathogen not only for hospital-acquired [1] and medical device-related infections [2, 3], but also for burn [4] and war-wound infections [5]. For such a wide spectrum of infections, the molecular pathogenesis is still incompletely understood. Mechanisms underlying the wide range of infections may entail the bacterial stress responses that help the microorganism to fit in new environments. The stress responses encompass the production of outer membrane vesicles (OMVs) in a process of vesiculation that occurs during all phases of growth of Gram-negative bacteria [6, 7]. OMVs also play a role in the pathogenesis as *P. aeruginosa *OMVs deliver multiple enzymes and virulence factors into the host cells [8]. Furthermore, bacteria produce OMVs in response to environmental and cellular stress factors [9–11], as vesiculation appears to increase survival of bacteria over stress. Environmental stress constitutes antibiotic treatments that have been found to affect vesiculation. Treatment of *Shigella dysenteriae* with mitomycin C, which activates the SOS response [12, 13], led to the increased level of Shiga toxin-associated OMV production [14] and toxin production [15]. Such stress-induced vesiculation seems to enhance survival, since the under-vesiculating mutants of *Escherichia coli *succumbed, whereas the overvesiculating mutants appeared more viable, when they were challenged with lethal envelope stressors [16]. With vesiculation identified as a bacterial stress response to environmental stimuli such as antibiotics, antibacterial treatment may aggravate the infections. Therefore, while mechanisms underlying vesiculation resulting from stress responses, especially the antimicrobial-triggered SOS response, remain poorly understood, it is imperative to investigate the connection so that effective intervention can be developed. 

**Figure 1:**
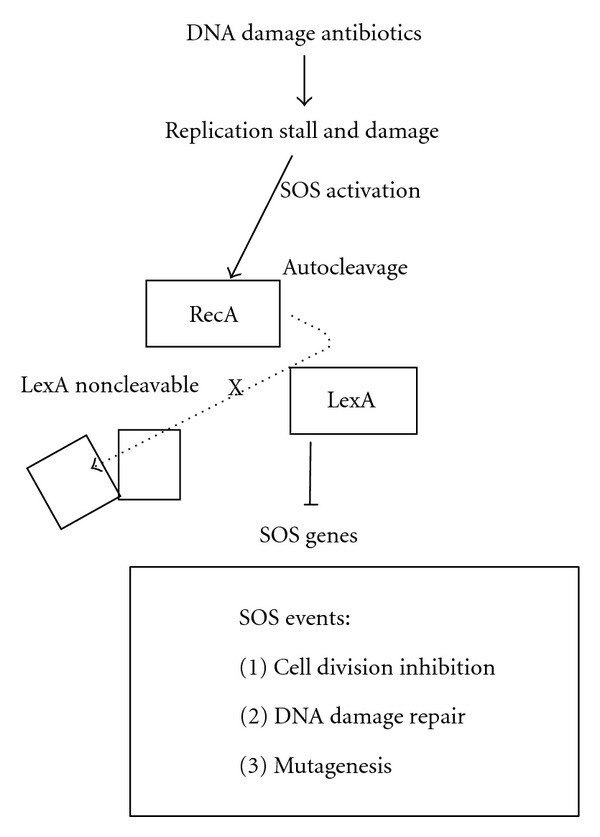
The bacterial SOS response. The response is triggered by DNA-damage antibiotics. This response is controlled by the RecA-LexA interplay, in which LexA represses the SOS genes. DNA damage activates RecA to simulate autocatalytic cleavage of LexA so that the SOS genes are depressed and expressed. X, the mutation rendering LexA noncleavable.

The SOS response [17] is a transcriptional response, in which LexA controls at least 40 SOS genes in *E. coli *[18–20] and 15 in *P. aeruginosa* [21]. SOS is triggered when bacteria are treated with DNA damage antibiotics, such as the quinolone antibiotic ciprofloxacin used in this work. The quinolone antibiotics target the type II topoisomerases including DNA gyrase (topoisomerase II) and topoisomerase IV [22]. These enzymes play an essential role in controlling superhelix density of chromosomal DNA to facilitate replication, recombination, repair, and transcription [22, 23]. Inhibiting these enzymes by ciprofloxacin leads to DNA strand breaks, the SOS signals. The mechanisms of the SOS response in* P. aeruginosa* and* E. coli* share the following steps (Figure 1). In the absence of the SOS signals, LexA blocks the transcription of the SOS genes [17]. When the SOS signals are generated during replication inhibition, RecA coprotease senses the signals and binds to the single-stranded DNAs to assume an active conformation [24]. Activated RecA stimulates the autocatalytic cleavage of LexA [25]. Consequently, LexA repression of the SOS genes is dismissed by this cleavage. Such derepression induces the SOS genes, leading to activation of the SOS response. One of them, *sulA,* is induced to inhibit and delay cell division transiently, resulting in cell filamentation, a sign of the SOS response, until DNA damage is ameliorated by the SOS proteins. The SOS proteins are involved in chromosome recombination, replication, repair, and segregation [26, 27]. As cell division is affected during SOS and is involved in OMV biogenesis [28], vesiculation may be linked to SOS. With antimicrobial agents inducing SOS and vesiculation, this link is quite likely as both SOS and vesiculation enhance bacterial survival [16, 29]. The purpose of this study is to investigate this link with multiple analyses. 

## 2. Materials and Methods

### 2.1. Bacterial Strains, Media, and Chemicals

*P. aeruginosa* PAO1 was obtained from the *Pseudomonas* Genetic Stock Center (strain PAO0001). The LexA noncleavable (*lexAN*) strain, gratefully from Dr. Floyd E. Romesberg, was constructed by the replacement of the catalytic serine of LexA with alanine as described in [21] so that the SOS regulon is repressed by *lexAN* in the presence of DNA damage. All the strains were grown at 37°C in Luria-Bertani (LB, purchased from Fisher Scientific) with 1-*μ*g/mL ciprofloxacin (Sigma-Aldrich, minimal inhibitory concentrations or MIC = 0.125 *μ*g/mL) as described before [21]. Experiments started with overnight cultures derived from the one-day-old single colonies grown on LB plates; experiments with colonies older than 3 days might not be reproducible.

### 2.2. Microscopic Analysis

Microscopy and measurement of cell length were performed as described [30, 31]. Briefly, the log-phase cells were fixed as described [30] and examined under a microscope (ZESS Axioshop 2 plus) equipped with CCD and computerized image analysis. The cell length was measured with NIH Image J. Significance levels (probability *P *values) in mean cell length were determined from a two-tailed Student's *t*-test.

### 2.3. OMV Extraction

Overnight cultures were diluted to the OD_600 nm_ of 0.01 with 7 mL LB in a 25-mL flat-bottom glass tube. The subcultures were grown in a shaker at 37°C at the 250-rpm speed for 8 hrs. After the first two hours, ciprofloxacin was added to the subcultures to the final concentration of 1 *μ*g/mL. OMVs were isolated by a standard method [32] with slight modifications. The cells in the subcultures were removed first by centrifugation at 10,000 rpm (12,000 g) for 10 minutes at 4°C and second by filtering the supernatant through a 0.2-*μ*m filter. The filtered supernatant (6 mL) was ultracentrifuged in a fixed angle rotor (Ti-1270) for 3 hours at 4°C for 33,000 rpm (100,000 g). The supernatant was discarded, and the pellet was resuspended in 50 *μ*L of phosphate buffered saline (PBS) by pipetting rather than by vortexing. The OMV samples could be stored at 4°C for 1 day for the macrophage assay and 3 days for proteomic analysis without losing activity.

### 2.4. Lipid Extraction and Quantification

The method was adopted from a protocol published previously [33] with slight modifications. The procedures included an extraction of the lipids with a mixture of methanol, chloroform, and water in a ratio of 2 : 2 : 0.8 (v/v). The OMV pellet collected after ultracentrifugation of the 7-mL cell-free culture was resuspended in 80 *μ*L water in an eppendorf tube and then, to the OMV suspension, 200 *μ*L methanol, and 200 *μ*L chloroform were added and mixed. To the cell pellet, water was added to the final volume of 500 *μ*L, and the cells were resuspended completely by vigorous vortexing. Then, an 80-*μ*L volume of cell suspension was transferred to an effendorf tube and mixed with 200 *μ*L methanol and 200 *μ*L chloroform. After a 10-min vortexing, the sample was centrifuged for 10 min at 13,000 rpm at 4°C. The chloroform layer was transferred completely by gently inserting a pipette through the water-methanol phase and the interphase down to the bottom of the tube. To the tube containing the aquatic phases, 100 *μ*L chloroform and 80 *μ*L water were added and mixed by a vortexing. Centrifugation and chloroform layer transfer were repeated as above. The two fractions of chloroform layers were pooled; effort should be made to avoid losing any volumes of these fractions. For quantification, an empty eppendorf tube had been weighed, and then, the chloroform fractions were transferred to it and evaporated to dryness in a speed vac. The dry lipids were weighed, and the net weight was acquired by subtracting the tube weight.

### 2.5. Transmission Electron Microscope (TEM)

TEM was conducted according to a standard protocol [34] with modifications. A 10-*μ*L volume of the OMV sample was placed on the lacey carbon film on 300-mesh copper grids. The grids were incubated at 25°C overnight. Then, they were negatively stained by 1% uranyl acetate (w/v, Sigma) for 20 seconds, washed three times with water, and air-dried. OMVs were examined on an Analytical Electron Microscope (JEOL JEM-2010F) with a Schottky field emission electron source. TEM was operated at accelerating voltage of 200 kV, with resolution at 0.1 nm lattice with 0.19 nm point-to-point, with magnification range from 2,0000x to 1,500,000x, with spot sizes of 2~5 nm at TEM mode, with EDS/NBD/CBD of 0.5~2.4 nm, and with specimen tilt at ±25 degrees (X, Y). Images were taken with a camera length of 80~2,000 mm and objective lens of Cs 0.5 mm and Cc 1.1 mm. For each sample, 5–10 images were recorded, and the diameter of each OMV was measured and compared for statistical significance as above.

### 2.6. Macrophage Cytotoxicity and MTT Assay

The indicated equal amount of OMVs (Figure 4) determined according to the OMV protein assay were suspended in DMEM medium and added into a 96-well plate which had been seeded with murine macrophage J774 cells (2 × 10^5^/well). The plate was incubated at 37°C with 5% CO_2_ (v/v). The cytotoxicity was examined in the macrophage cytotoxicity assays as described previously [35] with modifications. The seeded macrophage was incubated with OMVs at the indicated concentrations. First, macrophage morphology was examined under a phase-contrast microscope with a 40-x lens [VZEISS AXIOVERT-200 with AXIOCAM camera equipped with CCD and computerized image analysis] after 0.5, 1, 2, 4, and 24 h. Approximately 10 fields were examined for each of duplicate wells. Second, the commercial kit (cyto 96 nonradioactive cytotoxicity kits from Promega) was used to measure OMV cytotoxicity to macrophage. The concept of this kit is to measure a stable cytosolic enzyme lactate dehydrogenase (LDH), which is released when macrophage cells are lysed. The released LDH then reacts with NAD, and protons are donated to NAD from LDH. The reducing NADH then reacts with MTT [3-(4,5-dimethylthiazol-2-yl)-2,5-diphenyl tetrazolium bromide] and formazan (red) forms. By measuring the optical density of formazan at the wavelength of 490-nm, the cytotoxicity was quantified. The cytotoxicity is defined as the percentage of OMV-inflicted LDH release in contrast to the sum of detergent-lysed maximal release (positive control) and spontaneous release (negative control). The assay was carried out by following the manufacturer's protocol. The experiment wells were assembled to contain the 20-*μ*L OMV sample, the 80-*μ*L medium (DMEM plus 10% FBS, v/v and the 2 × 10^5^target cells J774 cells), and the OMV effectors. The following control wells were arranged. The wells for effecter spontaneous LDH release contained medium and OMVs. The wells for target cell spontaneous LDH release included only target cells (macrophage) and medium. The wells for target cell maximum LDH release carried medium, target cells, and the Lysis Solution. A 10-*μ*L volume of the Lysis Solution (10x) per 100 *μ*L of culture medium was added, and the mixture was incubated for 45 minutes before the supernatant was harvested. The wells for volume correction control contained only medium and lysis solution. The wells for culture medium background were used to correct phenol red and LDH activity that might be present in serum-containing culture medium. The 96-well plate then was incubated at 37°C with 5% CO_2_ (v/v) for 4 hours. After incubation, the supernatant (40–50 *μ*L) from each well (except for the Maximal LDH release control) was transferred into a well in a new 96-well plate and mixed with the same volume of substrate (tetrazolium salt, light-sensitive, so operate in dark). The 96-wells plate was incubated in the dark for 30 minutes at the room temperatures, and the reaction was terminated by adding the stop solution. The optical density was measured at the 490-nm wavelength.

### 2.7. OMV Proteomic Analysis

The proteomic analysis was performed by following a standard method [36] with slight modifications. Specifically, the OMV proteins were fractionated by SDS-PAGE [37] (10%, w/v) and stained by Coomassie blue. The lanes in replicates containing the proteins were cut out, sliced into pieces (1 × 1 mm), and placed into Eppendorf tubes. The slices were subjected to *in vitro* proteolysis by trypsin as follows. The first step was SDS removal from the gel slices. A 100-*μ*L volume of 25 mM NH_4_HCO_3_/50% acetonitrile (v/v, Fisher) was added to cover the gel slice. The mixture was vortexed for 10 minutes, and the supernatant was discarded. These steps were repeated until the gel became colorless. Acetonitrile (100%, v/v) was added to cover the gel slices, and the mixtures were incubated for a few minutes at room temperatures until the gel slices shrank and turned white. Acetonitrile in the gel slices was removed by spinning in a speed vacuum at room temperatures to complete dryness. The second step was reduction, alkylation, and proteolysis. For rehydration of the gel slices, an approximate 50-*μ*L volume of 10-mM DTT (Sigma) in 25-mM NH_4_HCO_3_ was added to each tube to cover the gel slices followed by vortexing and brief spinning. The reagents were allowed to react with gel pieces at 56°C for 60 minutes, and the supernatant was discarded. A 40-*μ*L volume of 55 mM iodoacetamide (Sigma) was added to the gel pieces, and the mixture was vortexed and then centrifuged briefly. The reaction was incubated in the dark for 30 min, and the supernatant was removed. The gels were washed in 100 *μ*L (or enough to cover the gels) of 25-mM NH_4_HCO_3_/50% acetonitrile (v/v) followed by vortex and centrifugation for 5 min. A 200-*μ*L volume of acetonitrile (100%, v/v) was added and removed as above. Trypsin (3 mg/mL, Promega) was added to just barely cover the gel pieces, and after a brief spinning, the mixture was incubated at 37°C for overnight. The third was extraction of peptides from the gel slices. The gel slices were centrifuged briefly, and the aquatic extract supernatant was collected into a 0.5-mL Eppendorf tube. To the gel pieces, a 30-*μ*L volume of 0.1% formic acid (v/v, Burdic & Jacson) in 25-mM NH_4_HCO_3_ was added, followed by vortexing for 15 min and spinning briefly. The supernatant was harvested and pooled into the aquatic extract supernatant. The collected samples were then spun in a speed-vacuum to reduce the volume to approximately 10 *μ*L (avoid complete dryness). The samples were stored at −20°C. Lastly, capillary liquid chromatography-tandem mass spectrometry (LC/MS/MS) was conducted at the RCMI Proteomics & Protein Biomarkers Cores to determine the peptides derived from the proteins in the gel slices. Capillary LC/MS/MS was performed with a linear ion trap tandem mass spectrometer (LTQ-XLS, ThermoFisher), where the top 7 eluting ions were fragmented by collision-induced dissociation.

Proteins were identified by searching MS/MS spectra against the NCBI nonredundant protein database (version 20100306; 10551781 sequences and 3596151245 residues). A probability-based database searching algorithm (Mascot, Matrixscience) was followed as described previously [38] with modifications. Briefly, database search criteria include taxonomy, bacteria (eubacteria, 3035644 sequences); enzyme, trypsin; variable modifications, carbamidomethylation of cysteines and oxidation of methionines; mass values, monoisotopic; protein mass, unrestricted; peptide mass tolerance, ±1000 ppm; fragment mass tolerance, ±0.8 Da; max missed cleavages, three instrument type, ESI-TRAP; number of queries, 87976. Peptide score distribution: Ions score is −10 log (*P*), where *P* is the probability that the observed match is a random event.

With the molecular weight search (MOWSE) peptide-mass database developed [39], the MOWSE scoring algorithm was used to calculate a score of each peptide entry. Briefly, the experimental mass values were searched across a calculated peptide mass database. Match of experimental mass values with calculated values were counted when the calculated value was in the range of a given mass tolerance of an experimental value. These matches were probability (*P*) based to ensure that the observed match is a random event. In a search for such random matches, the significance threshold was set for *P* to be ≤0.05, that is, a 1 in 20 chance of being a false positive. The matches were scored, based on the calculated *P*, that is, −10 log (*P*). The higher the score, the lower the *P* value. These ions scores were used to calculate protein score, which was the sum of the highest ions score for each distinct sequence. The proteins that were consistently detected in the replicates were counted. The inferred proteins were further categorized for functions and domains in amino acid sequences with the protein analysis software and with the published data. Functions and amino acid sequences were inferred by using http://www.uniprot.org/uniprot/O67077. Proteins with signal peptide were searched with http://www.signalpeptide.de/index.php?m=myproteinindex. Signal peptide in the proteins was predicted by using http://www.cbs.dtu.dk/services/SignalP/. Transmembrane domains were deduced with http://www.ch.embnet.org/software/TMPRED_form.html.

## 3. Results

### 3.1. Vesiculation Under Ciprofloxacin-Triggered SOS

**Figure 2:**
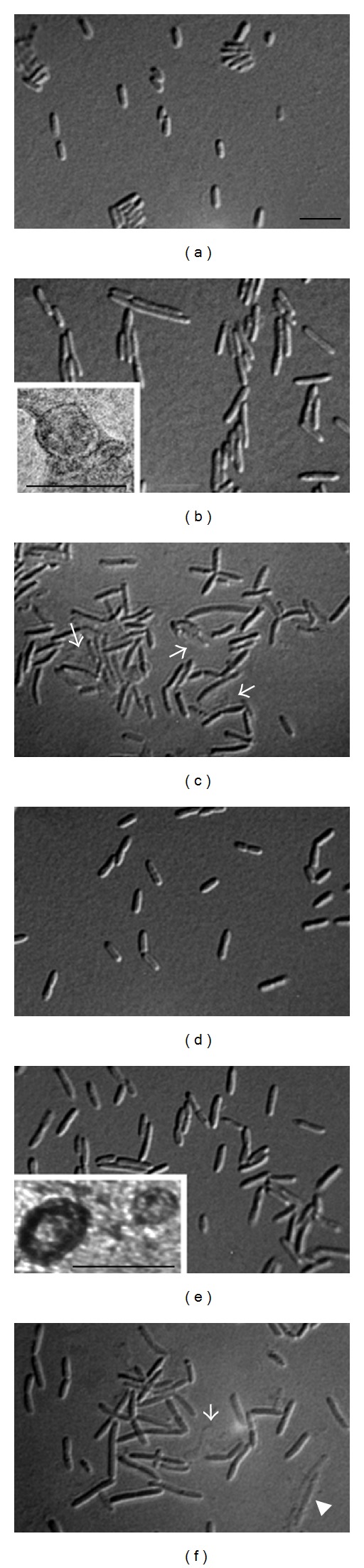
Microscopy of *P. aeruginosa *under the ciprofloxacininduced SOS response. The wild-type (PAO1) and the LexA noncleavable (*lexAN*) strains were grown in LB with shaking for 2 hrs, and then ciprofloxacin (CPX) was added (1 *μ*g/mL). The culture continued for 6 hrs. (a) PAO1 without and (b) with CPX. (c) PAO1 with 5 *μ*g/mL CPX. (d) *lexAN *without and (e) with CPX. (f) *lexAN* with 5 *μ*g/mL CPX. Arrows in (c) and (f) show damaged and lysed cells. Cell bar, 5 *μ*m. Inset (b) shows transmission electron microscopy of OMVs from the treated wild-type cultures. Inset (e) shows *lexAN *OMVs. OMV bar, 0.1 *μ*m.

The hypothesis concerning association of SOS with vesiculation was tested. The *P. aeruginosa *wild-type and the LexA noncleavable (*lexAN*) strains were treated with ciprofloxacin at 1 *μ*g/mL. OMVs were extracted from these strains. The rationale for the antibiotic treatment was that this antibiotic was known to activate the SOS response in *P. aeruginosa *at 1 *μ*g/mL, but SOS was noninducible in the *lexAN* strain [21]. Thus, testing of these strains with this drug would provide data relevant to SOS. When treated with the antibiotic, the wild-type cells became more filamented (cell length: 5.1 *μ*m ± 1.2 and *n* = 169) than the *lexAN* cells (4.61 *μ*m ± 1.2 and *n* = 89) (Figures 2(b) and 2(e), *P* < 0.0001). The significant cell filamentation is the manifestation of the SOS response [30, 40–43]. It is impossible to complement the *lexAN* mutant, because the *lexAN* phenotype is dominant; that is, in the *lexAN* background, the wild-type LexA would be cleaved, while the *lexAN* would remain not degraded during SOS. Besides, when both the wild-type and the *lexAN* cells were treated with ciprofloxacin at 1 *μ*g/mL, lysed cells appeared imperceptible (<2%, *n* = 500), in contrast to treatment at 5 *μ*g/mL (minimal bactericidal concentration, MBC = 3.25 *μ*g/mL) that led to noticeable damaged and lysed cells (20%–30%, Figures 2(c) and 2(f)). With the growth conditions determined, OMVs were isolated by ultracentrifugation from the cell-free supernatants of the wild-type and the *lexAN* cultures shown in Figures 2(b) and 2(e). The presence of OMVs in the samples was confirmed with transmission electron microscopy (Figures 2(b) and 2(e) insets). The diameters of OMVs from both strains appeared similar (*P* = 0.2 and *n* = 70). Additionally, phage activity was not detected in the 1-*μ*g/mL-drug-treated cell-free cultures and the OMV samples (data not shown). Hence, when the cells grew with the antibiotic at 1 *μ*g/mL and produced OMVs, the likelihood of OMV contamination with the unrelated proteins from lysed cells appeared very small and was further addressed as below.

### 3.2. Increase in OMV Protein Levels under SOS

**Figure 3:**
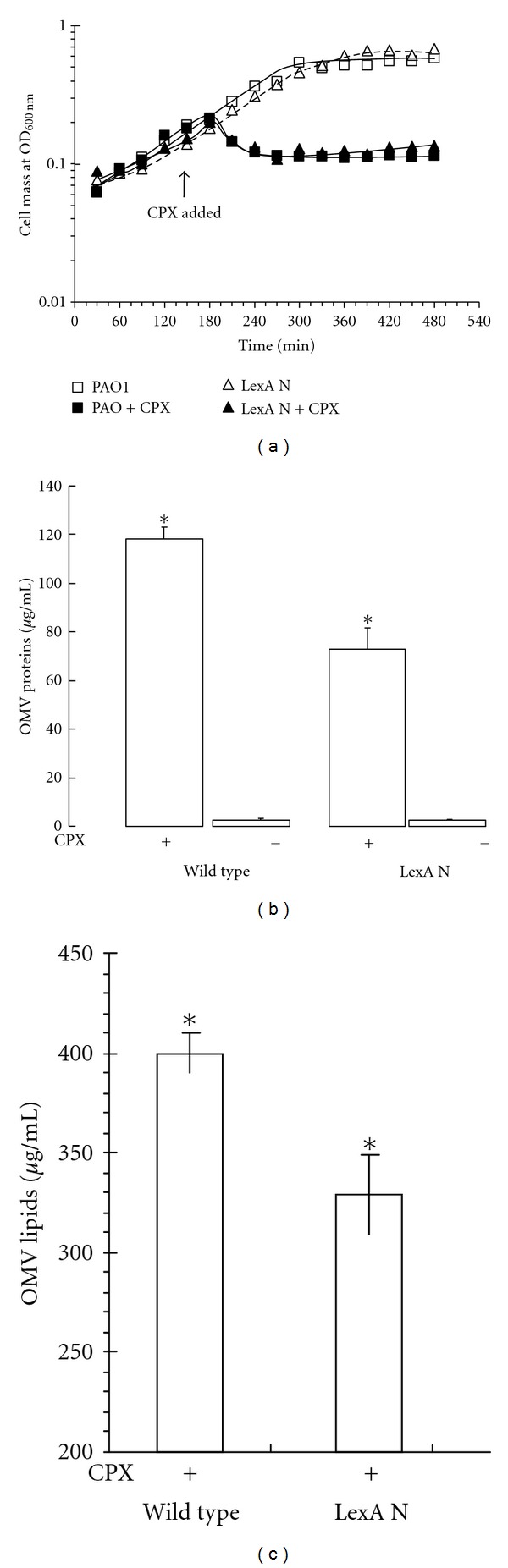
OMV production from *P. aeruginosa *under the ciprofloxacin-induced SOS response. (a) Growth of the wild-type (PAO1) and the LexA noncleavable strains. Both strain were grown in LB with shaking for 2 hrs, and then ciprofloxacin (CPX) was added. The culture continued for 6 hrs. OMVs were isolated from the wild-type and the *lexAN *cells treated with or without ciprofloxacin (CPX). (b) Quantification of OMV proteins by Bradford (*n* = 7) and (c) OMV lipids by weight (*n* = 3). (*P < 0.05).

OMVs were quantified from the wild-type and the *lexAN* strains treated with ciprofloxacin at 1 *μ*g/mL. Briefly, both the wild-type and LexA noncleavable strains were grown with ciprofloxacin at 1 *μ*g/mL. Both strains exhibited similar growth behaviors in the absence and in the presence of ciprofloxacin (Figure 3(a)). Since OMV protein quantity appeared to reflect the levels of OMVs [44], the OMV levels were determined from the same volume of culture containing the equal number of cells (Figure 3(a) at 480 min) by using Bradford assay of OMV proteins. With the ciprofloxacin treatment, the level of the wild-type OMV proteins increased more than 100-fold, as compared to that without (*P < 0.0001, Figure 3(a)). While the level of OMV proteins from the treated *lexAN* strain went up versus that of the untreated (*P* < 0.01), it did not reach the wild-type level, displaying 33% reduction reproducibly below the wild-type level (*P < 0.05, Figure 3(a)). These results demonstrate that vesiculation is stimulated by the antibiotic treatment. The data with *lexAN* suggest that the stimulation is attributed by LexA-dependent and independent mechanisms. The LexA-dependent mechanism of OMV stimulation involves SOS. The OMV protein level in the wild-type strain, which increased above that of the *lexAN* strain, was suppressed in the *lexAN* strain. Namely, the levels were increased when the SOS repressor LexA was autocleaved in the wild-type strain during SOS [25], but when LexA was made noncleavable in the *lexAN* strain [21], further augmentation appeared to cease. Therefore, these OMV proteins increased in the wild type but suppressed in the *lexAN* strain were termed the SOS-related. The LexA-independent mechanism may account for the increased levels of OMV proteins from the *lexAN* mutant over those from the untreated. These proteins were accordingly termed the SOS independent. Yet, the level was lower than that of the SOS-induced wild type (Figure 3(a)). In conclusion, the OMV protein level is increased from the cells treated with the antibiotic, and SOS contributes to the additional augmentation.

### 3.3. Increase in OMV Lipid Levels under SOS

It seemed possible that the proteins unrelated to OMVs but produced during SOS might be co-centrifuged with OMVs. This possibility was excluded by OMV lipid quantification. From OMV protein quantification, given the observation that the increase in the OMV protein level in the treated wild-type strain was suppressed in the treated *lexAN* strain (Figure 3(a)), we wanted to confirm the increase in vesiculation with the lipid assay. The lipids were extracted from OMVs and cells, and the total dry lipids were weighed as described previously [33]. The OMV lipids of the wild-type strain treated with ciprofloxacin were heavier than those of the *lexAN* strain (*P < 0.05, Figure 3(b)). In contrast, the weights of the total lipids from cells did not change significantly, irrespective of strains and treatment (*P* ≥ 0.1, data not shown). Thus, the OMV lipid mass increased for the treated wild type, and the augmentation was suppressed in the treated *lexAN* strain, the results consistent with the OMV protein quantification. This consistency ruled out the possibility of OMV contamination with unrelated proteins but supported the notion of SOS involvement in vesiculation, yet, the SOS-unrelated factors contributing to the increase cannot be excluded.

### 3.4. OMV-Mediated Macrophage Cytotoxicity under SOS

**Figure 4:**
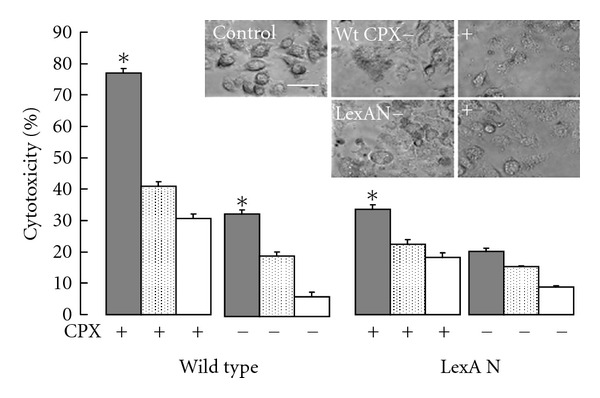
OMV-mediated macrophage cytotoxicity is SOS dependant. OMVs isolated from the wild-type (wt) and the LexA noncleavable (*lexAN*) cells as indicated were incubated with macrophage. Top: phase-contrast microscopy of macrophage incubated with OMVs at 1.3 *μ*g/mL after a 1 hr incubation. Bar, 20 *μ*m. Bottom: cytotoxicity was measured as the levels of released cytosolic lactate dehydrogenase after 4 hr incubation. It was set 0 for the macrophage-only control. OMV concentrations: grey, 1.3 *μ*g/mL; doted, 0.65 *μ*g/mL; blank, 0.325 *μ*g/mL. (*P < 0.05, *n* = 4).

Since OMVs act as a virulence factor [8, 45] and the OMV levels increase during the antibiotic-induced SOS, we wanted to investigate whether OMVs from the SOS strains differentially aggravates cytotoxicity. OMVs isolated from the wild-type and the *lexAN* cultures, either treated as above with ciprofloxacin or without, were added to macrophage in equal amounts. The cytotoxicity was assessed as described previously [35]. First, macrophage morphology was examined after incubation with OMVs from the untreated wild-type bacterial cells for 0.5, 1, 2, 4, and 24 h. Morphology changes appeared in the first h (Figure 4 Upper). OMVs from the ciprofloxacin-treated wild-type and the mutant cells caused dramatic alterations in the morphology of the macrophage, including cell shrinkage, detachment, and lysis, when compared with OMVs from the untreated bacterial cells. In contrast, treatment of macrophage with ciprofloxacin at 1 *μ*g/mL did not cause the cytotoxic morphology, the result excluding a possibility of ciprofloxacin-inflicted toxicity presumably caused by the drug-carrying OMVs. Second, the cytotoxicity was quantified. It is defined as the percentage of the OMV-inflicted LDH release from macrophage in the detergent-lysed maximal release from macrophage (OMVs or macrophage alone did not lead to a LDH-increase). As shown in Figure 4 (lower), the OMV-inflicted macrophage toxicity appeared concentration-dependent. Cytotoxicity by OMVs from the treated wild-type strain increased over 55% versus that by OMVs from the untreated (*P* < 0.05). These results indicate that the OMV-mediated cytotoxicity is stimulated by OMVs from the antibiotic-treated bacterial cells. However, this stimulated cytotoxicity was not observed in OMVs from the treated *lexAN* culture (*P* < 0.05). Therefore, LexA appeared to suppress the increased cytotoxicity. 

### 3.5. OMV Subproteomic Analysis

Under the ciprofloxacin treatment, the increased levels of the OMV proteins, lipids and the OMV-mediated cytotoxicity in the wild-type strain appeared to be suppressed in the *lexAN* strain. Many interesting questions were raised from these results as to what OMV proteins would be LexA-suppressed and what would be cytotoxicity-related. To address them, we examined the OMV subproteomes from the treated wild-type and the *lexAN* strains. The rationale for targeting the two treated strains was the following. For the treated wild-type strains, the OMV protein level was increased but suppressed for the *lexAN* strain. Thus, comparison of the OMV subproteomic data obtained from the two treated strains would provide information relevant to LexA or SOS. The comparison could help sort out the OMV proteins: the LexA-related and ciprofloxacin-specific or SOS-unrelated OMV proteins. When bacteria are treated with a certain antibiotic, drug-specific proteins were previously observed, such as OMPs [46, 47] and OMV proteins [48], which are unrelated to SOS. Most likely, the ciprofloxacin-specific OMV proteins would be found in both the wild-type and the *lexAN* OMV subproteomes, whereas the LexA-related OMV proteins would be detected in the OMV subproteome of the wild-type strain where LexA is autocleaved. 

**Figure 5:**
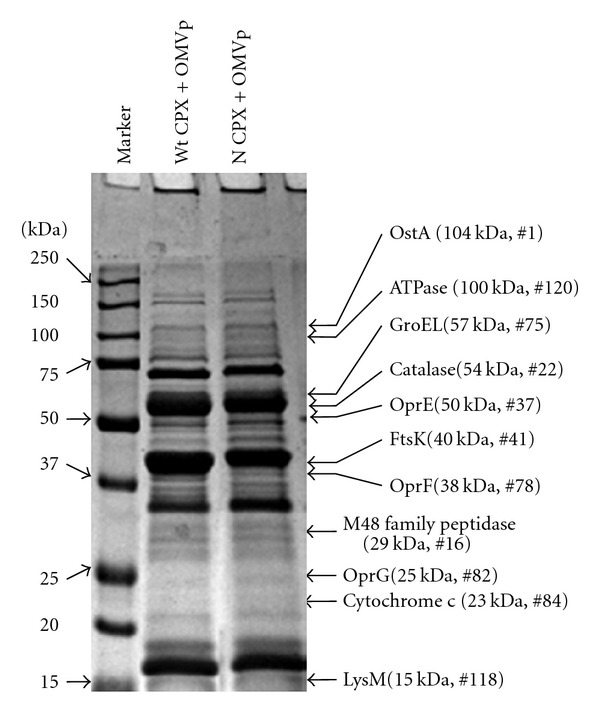
The SDS-PAGE based proteomic analyses of OMV proteins from the SOS-induced and the un-induced cells. CPX, ciprofloxacin. N, LexA noncleavable; and wt, wild-type. Illustrated to the right were the proteins known to associate with OMVs, cytotoxicity, and SOS (See text and Tables for details).

Experimentally, the OMV proteins from the antibiotic-treated wild-type and *lexAN* strains were characterized by the SDS-PAGE-based proteomic analysis (Figure 5). While small differences were observed in the OMV protein profiles for the treated wild-type and the *lexAN* strains (Figure 5), subtle distinctions were expected, based on LexA repression of gene expression, especially of the SOS regulons [18–21]. To unveil the differences, the *in vitro* trypsin proteolysis and capillary LC/MS/MS analysis was performed to determine the OMV proteins in the gel slices (Figure 5). The degraded peptide masses were determined and searched across the bacterial protein databases with the *P* < 0.05-based MOWSE scoring algorithm [39]. Totally, 145 proteins were identified in the OMV subproteomes from the treated wild-type and the *lexAN* strains (Tables 1–3). Many of the known OMV proteins, such as OstA (no. 1) [49] and OprE (no. 37) [50, 51], were detected in the wild-type OMVs (Figure 5 and Table 1), whereas GroEL (no. 75) [52] and OprF (no. 78) [50, 51] in both the wild-type and the *lexAN* OMVs (Table 2). Thus, the OMV subproteomes were confirmed to harbor some known OMV proteins. Moreover, with the SOS status of the wild-type and the *lexAN* strains used, the subproteomic analyses led to discovery and categorization of SOS- and cytotoxicity-related OMV proteins. The proteins detected only in OMVs from the drug-treated wild-type cells were termed the WT OMV proteins (74 proteins listed in Table 1 or 51% of 145). Since SOS was triggered in the wild type but repressed in the *lexAN* strain [21], the proteins produced during SOS were expected to emerge in the wild-type OMVs but not in the *lexAN* OMVs. These proteins were SOS-related. However, the proteins present in OMVs from both the treated wild-type and the *lexAN* strains were named the common OMV proteins (35 in Table 2 or 24%). The presence in both the OMV subproteomes implied that the appearance in OMVs was not affected by LexA; thus these proteins were called the SOS-unrelated. The OMV proteins present in OMVs from the *lexAN* cells alone were called the *lexAN* OMV proteins (36 in Table 3 or 26%). While the categorization provides insights into the antibiotic-stimulated vesiculation, it does not seem reconciled with the protein banding profiles that show slight differences in OMV proteins from the wild-type and the *lexAN* strains. The apparent discrepancy stems from the limited capacity of SDS-PAGE in resolving proteins with similar sizes in a certain band and inability to separate proteins of similar masses but of different pIs. For instance, when parallel bands in the wild-type and the *lexAN* OMV proteins were cut off for proteomic analysis, the subproteomic contents of the proteins carrying various pIs in one band were not completely identical to those in its counterpart (data not shown). Obviously, the OMV subproteomic analysis appears comprehensive, remedying the limitation of SDS-PAGE analysis. 

Interestingly, the OMV subproteomes seem to reflect the physiology of the cells under the antibiotic treatment and the cytotoxicity of OMVs to host cells. For instance, the known SOS-regulated proteins, such as FtsK (no. 41) [53] and catalase (no. 22) [54], were detected in the OMVs from the treated wild-type cells where SOS is induced. Proteins of efflux (no. 17) and cell motility (nos. 3, 34, and 42) were also found. Since efflux and cell mobility are involved in antibiotic resistance [55, 56], the presence of the related proteins in OMVs is likely to result from a response of the bacterial cells to the ciprofloxacin treatment. Furthermore, virulent proteins were detected in OMVs. In fact, cell mobility as mentioned above is known to enhance production of virulence factors [55]. An example in the WT OMV proteins is M48 family peptidase (no. 16 in Table 1) that contains *Pseudomonas* metalloproteases, elastase, and alkaline protease. These proteins are believed to mediate tissue penetration [57–59]. The examples in the common category include cytochrome c (no. 84 in Table 2) cytotoxic to macrophage [60], and OprG (no. 82) contributing to cytotoxicity toward human bronchial epithelial cells [61]. Examples in the *lexAN* group are the following: ATP-utilizing enzymes such as ATPase (no. 120 of Table 3) cytotoxic to macrophage [60] and the LysM domain carrying protein (no. 118) involved in pathogenesis [62]. Taken together, the OMV subproteomic results appeared aligned with the functional results pertinent to drug resistance, SOS, and cytotoxicity.

## 4. Discussion

Vesiculation from *P. aeruginosa* under ciprofloxacin treatment was investigated with multiple approaches. OMVs were isolated from the wild-type strain in which SOS is induced by ciprofloxacin and from the *lexAN* strain in which SOS is repressed. Cell morphology after the treatment showed cell filamentation, confirming SOS, while OMVs were not changed significantly in size during SOS. Vesiculation as determined chemically by the OMV protein and lipid levels and functionally by cytotoxicity is stimulated by the drug treatment, higher in the wild-type strain but suppressed in the *lexAN* strain. The overall increases for the wild-type and the *lexAN* strains suggest that the stimulation is attributed by the SOS-related and the independent factors; the suppression of further increase in the *lexAN* strain suggests that the additional augmentation involves SOS. The cytotoxicity of OMVs and the bacterial physiology under the antibiotic treatment and SOS were reflected by the results of the OMV subproteomic analysis. 

An intriguing observation is the presence of cytosolic proteins in OMVs. Considering the hydrophobic nature of outer membrane, we were tempted to suspect contamination of OMVs with the cytosolic proteins. Nevertheless, the presence of the cytosolic proteins in OMVs is not just coincidental but consistently documented [63]. In fact, GroEL (no. 75), ribosomal proteins (nos. 76, 121, and 124), and DNA binding proteins (no. 96, 115) were detected in outer membrane [52, 64–66] and OMV fractions [52, 67]. The possible mechanisms for their OMV inclusion may involve association of the cytoplasmic proteins with membrane proteins that may bring the former to membrane proximity. For example, peptidyl-prolyl *cis-trans* isomerase, a membrane-associated protein (no. 95 in Table 2), is a trigger factor that is highly conserved in most bacteria [68, 69]. The presence of the trigger factor in the stressed cells is reasonable as the trigger factor is generally believed to play a central role in bacterial survival of environmental insult. Since the trigger factor in *E. coli* is associated with the 50S ribosomal subunit [70] and GroEL [71–73], the factor is likely to be translocated with 50S ribosomal protein L28 and GroEL to OMVs. Besides, because OMVs can package DNA [48, 74] and *P. aeruginosa* OMVs carry DNA [75], the DNA binding proteins, such as DNA-binding stress protein (no. 115) and DNA-methyltransferase (no. 96), may be delivered into OMVs through hitching onto DNA. 

The molecular mechanisms behind the vesiculation stimulation during SOS remain poorly understood. OMVs are generated from living cells by budding from outer membrane bulges with subsequent fission [6, 7, 48, 74, 76]. Vesiculation does not concur with cell lysis, for OMVs package newly synthesized proteins [77–79]. These may be the reasons that OMV yields were too low when ciprofloxacin was used at and above MBC (data not shown), but the OMV levels were high when the drug was administered at 1 *μ*g/mL. Therefore, the increase in the OMV protein levels observed in this work from the cultures treated with ciprofloxacin at 1 *μ*g/mL is unlikely to result from cell lysis, especially as lysed cells barely were observed in the culture treated with the drug. The OMV protein levels are most likely to reflect the vesiculation stimulation during SOS. Indeed, the SOS-induced vesiculation is corroborated by the OMV lipid quantification. The OMV induction can be interpreted by combination of cell division delay and envelope alteration incurred in SOS. During SOS, *sulA* is induced, whose product inhibits and delays cell division transiently until DNA damage is ameliorated. In *E. coli, *this is achieved by SulA binding to FtsZ to block septum formation [42, 43, 80, 81]; similarly, a complex of *P. aeruginosa* SulA with FtsZ has been reported [82]. Inhibition of cell division was observed in *P. aeruginosa* treated with ciprofloxacin (Figure 2). According to the model of OMV biogenesis [28], such an episode of division inhibition may invoke temporary impact on the envelope structure, stimulating OMV generation. 

Our finding of vesiculation stimulation during SOS is highly significant. On one hand, suppression of the SOS-repair network by LexA in *E. coli* with engineered bacteriophage increased bactericidal effects of SOS-inducing antibiotics *in vitro* and enhanced survival of infected mice *in vivo *[83], paving a way for the LexA-based therapeutic strategy. In parallel are our results that LexA represses OMV stimulation and cytotoxicity, yet the *lexAN*-based strategy fails to eliminate them (Figures 3 and 4), pointing to existence of LexA independent mechanisms. The OMV protein levels increased in the cultures of the ciprofloxacin-treated *lexAN* mutant; the noncleavable LexA even appeared to contribute to production of some OMV proteins (Figure 3(a) and Table 3). The observations lead to the SOS-independent mechanisms that demand future endeavor in investigation. On the other hand, SOS appears responsible for the antibiotic inducible-biofilm formation [30, 40] and vesiculation though the mechanisms behind the induction seem a mystery. Our data obtained with the OMV protein-, the lipid-, the cell- and the proteomic-based approaches suggest that SOS plays a role in the antibiotic-stimulated vesiculation and in OMV-mediated cytotoxicity to macrophage. The result may help develop guidelines for antibiotic practice to prevent such side effects as vesiculation and the related cytotoxicity to host defense cells.

## Figures and Tables

**Table 1:** The WT OMV proteins.

Protein access ID	Protein description	Score	pI	Mass	Predicted functions	Membrane domains
(1) gi∣15595792	OstA precursor [*P. aeruginosa *PAO1]	366	5.41	104207	Organic solvent tolerance protein	SP, TM
(2) gi∣30525581	ChaPs, heat-shock protein [*Piscirickettsia salmonis*]	174	4.79	57310	Protein folding, immunogenic protein	n, TM
(3) gi∣15595608	PilJ [*P. aeruginosa* PAO1]	165	4.65	72484	Cell motility, twitching motility	SP, TM
(4) gi∣15597641	Glycine dehydrogenase [*P. aeruginosa* PAO1]	133	5.68	103853	Degradation of glycine	n, TM
(5) gi∣254236694	OprC precursor, putative [*P. aeruginosa* C3719]	117	6.02	79268	Copper transport outer membrane porin	SP, TM
(6) gi∣15599015	SecF [*P. aeruginosa *PAO1]	115	4.81	33021	Protein translocation	SP, TM
(7) gi∣15599417	Fe(III)-pyochelin OM receptor precursor [*P. aeruginosa *PAO1]	107	5.86	79943	Siderophore-iron transmembrane transporter activity	SP, TM
(8) gi∣309885	Aspartate transcarbamoylase [*P. aeruginosa* PAO1]	102	6.17	36521	Pyrimidine biosynthesis	n, n
(9) gi∣83718562	Peptide synthetase-like protein [*Burkholderia thailandensis *E264]	96	5.91	349402	Peptide synthesis	n, TM
(10) gi∣152986344	YaeT [*P. aeruginosa *PA7]	94	5.02	88222	OMP assembly complex	SP, TM
(11) gi∣124022818	Phosphate binding protein [*Prochlorococcus marinus* str. MIT 9303]	90	9.51	18989	ABC transport	SP, TM
(12) gi∣167840097	Phospholipase D [*Burkholderia thailandensis* MSMB43]	88	6	64431	Lipid catalytic activity	n, TM
(13) gi∣167035883	TonB-dependent copper receptor [*P. putida* GB-1]	87	5.77	74399	Copper receptor	SP, TM
(14) gi∣28871621	Polyribonucleotide nucleotidyltransferase [*P. syringae pv. tomato* str. DC3000]	81	5.20	74880	mRNA degradation	n, TM
(15) gi∣15600748	ATP synthase subunit *γ* [*P. aeruginosa* PAO1]	73	7.70	31533	ATP production	n,n
(16) gi∣70732441	M48 family peptidase [*P. fluorescens *Pf-5]	69	7.66	28833	Protein dagradation	SP, TM
(17) gi∣50085971	Acridine efflux pump [*Acinetobacter *sp. ADP1]	67	6.36	114705	Efflux pump	n, TM
(18) gi∣15596751	Cytochrome oxidase subunit (cbb3-type) [*P. aeruginosa *PAO1]	66	9.42	53073	Electron transport chain	n, TM
(19) gi∣119713613	SecD [uncultured marine bacterium EB0_39H12]	62	5.63	67763	Protein translocation	n, TM
(20) gi∣7715581	PspA [*Streptococcus pneumoniae*]	61	4.70	27305	Pneumococcal surface protein A	n, n
(21) gi∣10945103	PapA [*P. aeruginosa*]	61	4.87	71649	Lipid metabolic process	SP, TM
(22) gi∣121605556	Catalase [*Polaromonas naphthalenivorans *CJ2]	61	6.26	54212	Hydrogen peroxide catabolic process	n, n
(23) gi∣94495248	Rhizopine catabolism protein [*Sphingomonas* sp. SKA58]	60	5.91	38825	Oxidation reduction	n, TM
(24) gi∣116328093	Zn-dependent hydrolase [*Leptospira borgpetersenii* serovar Hardjo-bovis L550]	59	5.67	30997	Hydrolase activity	SP, TM
(25) gi∣149378317	PAS [*Marinobacter algicola* DG893]	59	5.97	57059	Signal transducer activity, chemotaxis	TM
(26) gi∣119356290	Molybdate ABC transporter, ATPase subunit [*Chlorobium phaeobacteroides* DSM 266]	59	9.23	38876	ABC transport	n, n
(27) gi∣117619653	Methyl-accepting chemotaxis transducer [*P. stutzeri* A1501]	59	4.67	58670	Methyl-accepting chemotaxis	SP, TM
(28) gi∣119899764	MCP-domain-containing signal transduction protein [*Azoarcus* sp. BH72]	59	5.80	53615	Signal transduction	SP, TM
(29) gi∣887858	DcrH [*Desulfovibrio vulgaris* str. Hildenborough]	59	5.71	104664	Signal transduction	SP, TM
(30) gi∣28869218	Aerotaxis receptor [*P. syringae* pv. tomato str. DC3000]	59	5.70	56965	Aerotaxis	n, TM
(31) gi∣112004994	Symbionin [*Buchnera aphidicola*]	58	5.13	57904	Protein folding	n, TM
(32) gi∣15597430	PslD, biofilm proteins [*P. aeruginosa PAO1*]	58	8.66	27891	polysaccharide transmembrane transport	SP, TM
(33) gi∣154253615	Molybdenum cofactor biosynthesis protein C [*Parvibaculum lavamentivorans *DS-1]	58	9.64	16871	Cofactor biosynthesis	n, n
(34) gi∣15600235	PilO [*P. aeruginosa *PAO1]	57	5.04	22805	Cell motility, type 4 fimbrial biogenesis	n, TM
(35) gi∣29830660	Oxidoreductase [*Streptomyces avermitilis* MA-4680]	57	5.01	33162	Electron transport chain	n, TM
(36) gi∣108803685	Bifunctional homocysteine S-methyltransferase [*Rubrobacter xylanophilus* DSM 9941]	57	6.33	65183	Amino-acid biosynthesis	n, TM
(37) gi∣15595488	OprE precursor [*P. aeruginosa PAO1*]	54	8.67	49637	Anaerobically-induced OM porin	SP, TM
(38) gi∣121997032	Glycolate oxidase iron-sulfur subunit [*Halorhodospira halophila* SL1]	53	8.55	43945	Oxidation reduction	n, n
(39) gi∣161508096	Biotin-acetyl-CoA-carboxylase ligase [*Lactobacillus helveticus* DPC 4571]	53	9	37120	Protein modification process	n, n
(40) gi∣119773234	GTPase EngB [*Shewanella amazonensis* SB2B]	52	6.97	24168	Cell division	n, n
(41) gi∣75764772	FtsK [*Bacillus thuringiensis *serovar israelensis ATCC 35646]	50	4.33	40378	Cell division	SP, TM
(42) gi∣3237312	FimV [*P. aeruginosa* PAO1]	50	4.33	98113	Cell motility, twitching motility	SP, TM
(43) gi∣145588417	Ferric uptake regulator family protein [*Polynucleobacter necessarius* subsp. asymbioticus QLW-P1DMWA-1]	50	6.29	17176	Repressor of the iron transport operon	n, n
(44) gi∣163846285	Deoxyribose-phosphate aldolase [*Chloroflexus aurantiacus* J-10-fl]	50	6.08	27440	Deoxyribonucleotide catabolic process	n, n
(45) gi∣107099581	Hypothetical+C448_01000594 [*P. aeruginosa *PACS2]	267	5.13	101779	Unknown	Unknown
(46) gi∣15598924	Hypothetical PA3729 [*P. aeruginosa PAO1*]	249	5.18	75836	Unknown	n, TM
(47) gi∣15599685	Hypothetical PA4489 [*P. aeruginosa PAO1*]	179	5.48	167326	Putative endopeptidase inhibitor activity	SP, TM
(48) gi∣156932378	Hypothetical ESA_00154 [Enterobacter sakazakii ATCC BAA-894]	167	4.83	57278	Protein folding	n, TM
(49) gi∣15596261	Hypothetical PA1064 [*P. aeruginosa *PAO1]	116	5.38	24157	Unknown	n, TM
(50) gi∣218890269	Hypothetical PLES_15291 [*P. aeruginosa *LESB58]	97	6.34	28636	Unknown	SP, TM
(51) gi∣15595825	Hypothetical PA0628 [*P. aeruginosa *PAO1]	92	8.80	35848	Unknown	n, n
(52) gi∣15600425	Hypothetical PA5232 [*P. aeruginosa* PAO1]	90	9.11	38586	Protein transporter	SP, TM
(53) gi∣15596143	Hypothetical PA0946 [*P. aeruginosa* PAO1]	86	4.93	36754	Unknown	SP, TM
(54) gi∣107103341	Hypothetical PaerPA_01004410 [*P*. aeruginosa PACS2]	84	9.02	67631	Unknown	Unknown
(55) gi∣15599691	Hypothetical PA4495 [*P. aeruginosa *PAO1]	84	5.79	24864	Unknown	SP, TM
(56) gi∣15600607	Hypothetical PA5414 [*P. aeruginosa *PAO1]	75	5.76	22533	Unknown	SP, TM
(57) gi∣1162960	Protein homologous to HI0366 in Haemophilus influenzae [*P. aeruginosa *PAO1]	72	6.77	22403	Unknown	SP, TM
(58) gi∣148980343	Hypothetical VSWAT3_23674 [*Vibrionales bacterium* SWAT-3]	59	4.85	56161	Chemotaxis	SP, TM
(59) gi∣86146473	Hypothetical MED222_12698 [*Vibrio* sp. MED222]	59	4.76	68870	Signal transducer activity	n, TM
(60) gi∣32266390	Hypothetical HH0891 [*Helicobacter hepaticus *ATCC 51449]	59	4.99	52013	Signal transducer activity	n, n
(61) gi∣89893845	Hypothetical DSY1099 [*Desulfitobacterium hafniense* Y51]	59	5.00	54148	Signal transducer activity	n, n
(62) gi∣18309647	Hypothetical CPE0665 [*Clostridium perfringens *str. 13]	59	4.91	28888	Unknown	Unknown
(63) gi∣160938898	Hypothetical CLOBOL_03792 [*Clostridium bolteae* ATCC BAA-613]	59	4.79	60828	Chemotaxis	SP, TM
(64) gi∣167772543	Hypothetical ANACOL_03921 [*Anaerotruncus colihominis* DSM 17241]	59	4.66	71478	Signal transducer activity	SP, TM
(65) gi∣158334828	Hypothetical AM1_1665 [*Acaryochloris marina* MBIC11017]	59	4.54	28589	Unknown	n, n
(66) gi∣146298741	Hypothetical Fjoh_0980 [*Flavobacterium johnsoniae* UW101]	58	8.90	168226	Unknown	SP, TM
(67) gi∣126348240	Conserved hypothetical [*Streptomyces ambofaciens* ATCC 23877]	58	4.68	123551	Unknown	Unknown
(68) gi∣162454210	Hypothetical sce5933 [*Sorangium cellulosum* So ce 56]	56	10.06	49450	Unknown	SP, TM
(69) gi∣159184340	Hypothetical Atu0493 [*Agrobacterium tumefaciens* str. C58]	56	8.83	17570	Unknown	Unknown
(70) gi∣120536835	Hypothetical Maqu_4123 [*Marinobacter aquaeolei* VT8]	54	6.02	36805	Unknown	n, n
(71) gi∣88800650	Hypothetical MED297_05259 [*Reinekea* sp. MED297]	53	5.05	74044	Unknown	Unknown
(72) gi∣107103648	Hypothetical PaerPA_01004718 [*P. aeruginosa* PACS2]	52	8.92	52714	Unknown	Unknown
(73) gi∣124385398	Hypothetical BMA10229_A0995 [*Burkholderia mallei* NCTC 10229]	52	5.66	5192	Unknown	n, n
(74) gi∣94986987	Hypothetical LI0545 [*Lawsonia intracellularis *PHE/MN1-00]	51	6.43	11768	Unknown	n, n

TM: transmembrane domains; SP: signal peptide; pI: isoelectric point.

**Table 2:** The common OMV proteins.

Protein access ID	Protein description	Score	pI	Mass	Predicted functions	Membrane domains
(75) gi∣576779	GroEL [*P. aeruginosa*]	784	5.04	57036	Protein folding	n, n
(76) gi∣167855908	50S ribosomal protein L28 [*Haemophilus parasuis* 29755]	242	4.90	57645	Protein synthesis	n, n
(77) gi∣15596780	Succinate dehydrogenase flavoprotein subunit [*P. aeruginosa *PAO1]	175	6.04	63492	Tricarboxylic acid cycle	n, TM
(78) gi∣15596974	OprF precursor [*P. aeruginosa* PAO1]	166	4.98	37616	Major porin, ion transport	SP, TM
(79) gi∣15596375	OprH precursor [*P. aeruginosa* PAO1]	107	9.00	21561	Response to Mg^2+^ starvation	SP, TM
(80) gi∣15598888	OMP precursor [*P. aeruginosa* PAO1]	103	9.45	28497	OM	SP, TM
(81) gi∣2626833	Chemotactic transducer [*P. aeruginosa*PAO1]	98	4.88	68395	Chemotaxis	SP, TM
(82) gi∣15599262	OprG precursor [*P. aeruginosa* PAO1]	93	4.85	25178	OmpW family	SP, TM
(83) gi∣15598278	Glycine betaine transmethylase [*P. aeruginosa* PAO1]	74	4.74	71360	Utilization of choline and glycine betaine as carbon and nitrogen sources	SP, TM
(84) gi∣15596750	Cytochrome c oxidase subunit [*P. aeruginosa* PAO1]	74	7.79	22744	Electron carrier activity	n. TM
(85) gi∣37522034	Glycosyltransferase [*Gloeobacter violaceus* PCC 7421]	70	8.96	47611	Biosynthesis of glycoproteins	n, n
(86) gi∣15596166	TolQ [*P. aeruginosa* PAO1]	67	5.96	25266	Import of group A colicins for envelope integrity	n, TM
(87) gi∣15599941	SecG [*P. aeruginosa* PAO1]	65	5.21	13199	Protein translocation	n, TM
(88) gi∣151545	RNA polymerase subunit [*P. aeruginosa*]	63	4.95	30372	RNA synthesis	n, n
(89) gi∣115523809	OmpA/MotB domain-containing protein [*RhodoP. palustris* BisA53]	63	7.60	45685	Major nonspecific porin	SP, TM
(90) gi∣114563330	Phosphoglucomutase [*Shewanella frigidimarina* NCIMB 400]	62	5.39	62288	Carbohydrate metabolic process	n, n
(91) gi∣15596170	OprL precursor [*P. aeruginosa *PAO1]	61	5.95	17914	Peptidoglycan-associated OM lipoprotein	SP, TM
(92) gi∣15598193	Na(+)-translocating NADH-quinone reductase subunit C [*P. aeruginosa* PAO1]	61	5.67	27763	Reduction of ubiquinone-1 to ubiquinol and transport of Na^+^ ions	SP, TM
(93) gi∣15598107	TonB-dependent receptor, putative [*P. aeruginosa* PAO1]	60	6.24	80241	High-affinity binding and energy-dependent uptake of specific substrates into the periplasmic space	SP, TM
(94) gi∣162455126	Protein kinase [*Sorangium cellulosum* So ce 56]	57	6.16	189825	Kinase activity	n, n
(95) gi∣15597002	Peptidyl-prolyl cis-trans isomerase D [*P. aeruginosa* PAO1]	57	4.99	68699	Protein folding	n, TM
(96) gi∣183602700	Site-specific DNA-methyltransferase [*Bifidobacterium animalis* subsp. lactis HN019]	56	6.24	51123	DNA methylation	n, n
(97) gi∣15600134	HflC [*P. aeruginosa* PAO1]	50	9.48	33095	Peptidase activity	SP, TM
(98) gi∣15599627	Iron-sulfur protein [*P. aeruginosa* PAO1]	50	6.07	20815	Iron-sulfur cluster assembly	n, n
(99) gi∣15595268	Hypothetica PA0070 [*P. aeruginosa* PAO1]	114	8.93	31697	Unknown	SP, TM
(100) gi∣15596030	Hypothetica PA0833 [*P. aeruginosa *PAO1]	104	8.89	24698	OmpA family	SP, TM
(101) gi∣15597431	Hypothetica PA2235 [*P. aeruginosa* PAO1]	93	5.99	74519	Lipopolysaccharide biosynthetic process	n, TM
(102) gi∣15595823	Hypothetica PA0626 [*P. aeruginosa* PAO1]	87	9.67	31273	Unknown	n, n
(103) gi∣15599828	Hypothetica PA4632 [*P. aeruginosa *PAO1]	81	6.97	29141	Proteolysis	SP, TM
(104) gi∣15599183	Hypothetica PA3988 [*P. aeruginosa* PAO1]	67	5.23	22870	OM assembly	SP, TM
(105) gi∣152989513	Hypothetica PSPA7_0777 [*P. aeruginosa* PA7]	62	4.77	17484	Unknown	n, n
(106) gi∣15595830	Hypothetica PA0633 [*P. aeruginosa *PAO1]	62	4.93	17528	Unknown	n, n
(107) gi∣15595812	Hypothetica PA0615 [*P. aeruginosa* PAO1]	60	4.53	18939	Unknown	n, n
(108) gi∣15595813	Hypothetica PA0616 [*P. aeruginosa* PAO1]	58	5.93	19410	Unknown	n, TM
(109) gi∣15599619	Hypothetica PA4423 [*P. aeruginosa* PAO1]	51	6.69	65589	Unknown	SP, TM

TM: transmembrane domains; SP: signal peptide. pI: isoelectric point.

**Table 3:** The *lexAN* OMV proteins.

Protein access ID	Protein description	Score	pI	Mass	Predicted functions	Membrane domains
(110) gi∣15599248	6,7-dimethyl-8-ribityllumazine synthase [*P. aeruginosa* PAO1]	299	5.69	16403	Riboflavin biosynthesis	n, n
(111) gi∣14573303	PilA [*P. aeruginosa*]	160	6.23	15488	Major pilin subunit of type IV pili	n, TM
(112) gi∣15598049	OprI precursor [*P. aeruginosa* PAO1]	132	7.90	8829	OM lipid-anchor.	SP, TM
(113) gi∣15596004	AmpDh3 [*P*. aeruginosa PAO1]	129	5.89	28703	Peptidoglycan catabolic process	n, n
(114) gi∣15599856	Lipid A 3-O-deacylase [*P. aeruginosa* PAO1]	116	5.87	18382	Modification of lipid A of LPS	SP, TM
(115) gi∣15596159	DNA-binding stress protein [*P. aeruginosa* PAO1]	100	4.96	17482	Response to stress, iron ion homeostasis	n, n
(116) gi∣15599000	PilF [*P. aeruginosa* PAO1]	75	6.67	28520	Type 4 fimbrial biogenesis	SP, TM
(117) gi∣15596133	LpxO2 [*P. aeruginosa* PAO1]	72	9.90	35737	LPS biosynthesis	n, TM
(118) gi∣15600371	LysM domain/BON superfamily protein [*P. aeruginosa *PAO1]	69	5.45	15451	Cleavage of septal peptidoglycan to allow cell separation	n, n
(119) gi∣15598026	HtpX [*P. aeruginosa* PAO1]	65	7.03	31573	Heat shock protein, proteolysis	SP, TM
(120) gi∣15600018	Mg(2+) transport ATPase, P-type 2 [*P. aeruginosa* PAO1]	63	5.88	99987	Magnesium-importing ATPase activity	n, TM
(121) gi∣15599764	50S ribosomal protein L21 [*P. aeruginosa* PAO1]	62	9.85	11646	Protein synthesis	n, n
(122) gi∣116620118	HAD family hydrolase [*Solibacter usitatus* Ellin6076]	57	5.37	22870	Phosphoglycolate phosphatase activity	n, n
(123) gi∣21233204	RhlB [*Xanthomonas campestris* pv. campestris str. ATCC 33913]	51	9.25	62279	ATP-dependent RNA helicase unwinding of double stranded RNA	n, n
(124) gi∣15599463	30S ribosomal protein S7 [*P. aeruginosa* PAO1]	51	10.24	17493	Protein synthesis	n, n
(125) gi∣73537822	Twin-arginine translocation pathway signal [*Ralstonia eutropha* JMP134]	50	9.37	36128	Protein export through the cytoplasmic membrane	SP, TM
(126) gi∣71064880	GltI [*Psychrobacter arcticus* 273-4]	50	5.11	35348	ABC glutamate/aspartate transporter	SP, n
(127) gi∣15596250	HypotheticalPA1053 [*P. aeruginosa *PAO1]	279	9.64	15639	Unknown	SP, TM
(128) gi∣15599835	HypotheticalPA4639 [*P. aeruginosa* PAO1]	98	9.47	20723	Unknown	SP, TM
(129) gi∣15600165	HypotheticalPA4972 [*P. aeruginosa* PAO1]	86	5.98	27836	Unknown	SP, TM
(130) gi∣15598227	HypotheticalPA3031 [*P. aeruginosa *PAO1]	83	4.92	8007	Unknown	SP, TM
(131) gi∣15598151	HypotheticalPA2955 [*P. aeruginosa* PAO1]	70	5.35	23677	Unknown	SP, TM
(132) gi∣145635820	HypotheticalCGSHiAA_01062 [*Haemophilus influenzae* PittAA]	69	5.85	56041	Unknown	n, n
(133) gi∣183222376	Hypothetical LEPBI_I3030 [*Leptospira biflexa serovar* Patoc strain Patoc 1 (Paris)]	61	8.40	51147	Transporter activity	SP, TM
(134) gi∣57233652	HypotheticalDET1586 [*Dehalococcoides ethenogenes* 195]	61	8.80	21290	Unknown	SP, TM
(135) gi∣29377408	HypotheticalEF2944 [*Enterococcus faecalis* V583]	58	4.99	19312	Unknown	n, n
(136) gi∣15597823	HypotheticalPA2627 [*P. aeruginosa *PAO1]	57	10.35	23048	Unknown	n, n
(137) gi∣116750341	HypotheticalSfum_2918 [*Syntrophobacter fumaroxidans* MPOB]	54	5.40	89798	Carbohydrate binding	SP, TM
(138) gi∣167751139	Hypothetical EUBSIR_02124 [*Eubacterium siraeum* DSM 15702]	53	5.50	44267	Metal ion binding	n, TM
(139) gi∣26250264	Hypotheticalc4442 [*E. coli* CFT073]	53	9.39	39090	Unknown	n, TM
(140) gi∣167754381	Hypothetical ALIPUT_02675 [*Alistipes putredinis* DSM 17216]	53	6.89	18845	Methyltransferase activity	n, n
(141) gi∣116048834	Hypothetical PA14_52490 [*P. aeruginosa* UCBPP-PA14]	52	6.19	17828	Unknown	n, n
(142) gi∣83312716	Hypothetical amb3617 [*Magnetospirillum magneticum* AMB-1]	52	5.42	89219	Cyclic nucleotide biosynthetic process	n, TM
(143) gi∣154503183	Hypothetical RUMGNA_01007 [*Ruminococcus gnavus* ATCC 29149]	51	5.29	49010	Rhamnose metabolic process	n, n
(144) gi∣15598505	Hypothetical PA3309 [*P. aeruginosa* PAO1]	51	5.50	16486	Response to stress	n, n
(145) gi∣148255416	HypotheticalBBta_4029 [*Bradyrhizobium* sp. BTAi1]	51	4.22	4004	Unknown	n, n

TM: transmembrane domains; SP: signal peptide. pI: isoelectric point.
